# Two-dimensional superconducting MoSi_2_N_4_(MoN)_4n_ homologous compounds

**DOI:** 10.1093/nsr/nwac273

**Published:** 2022-11-28

**Authors:** Zhibo Liu, Lei Wang, Yi-Lun Hong, Xing-Qiu Chen, Hui-Ming Cheng, Wencai Ren

**Affiliations:** Shenyang National Laboratory for Materials Science, Institute of Metal Research, Chinese Academy of Sciences, Shenyang 110016, China; Shenyang National Laboratory for Materials Science, Institute of Metal Research, Chinese Academy of Sciences, Shenyang 110016, China; School of Materials Science and Engineering, University of Science and Technology of China, Shenyang 110016, China; Shenyang National Laboratory for Materials Science, Institute of Metal Research, Chinese Academy of Sciences, Shenyang 110016, China; School of Materials Science and Engineering, University of Science and Technology of China, Shenyang 110016, China; Shenyang National Laboratory for Materials Science, Institute of Metal Research, Chinese Academy of Sciences, Shenyang 110016, China; School of Materials Science and Engineering, University of Science and Technology of China, Shenyang 110016, China; Shenyang National Laboratory for Materials Science, Institute of Metal Research, Chinese Academy of Sciences, Shenyang 110016, China; School of Materials Science and Engineering, University of Science and Technology of China, Shenyang 110016, China; Shenzhen Institute of Advanced Technology, Chinese Academy of Sciences, Shenzhen 518055, China; Shenyang National Laboratory for Materials Science, Institute of Metal Research, Chinese Academy of Sciences, Shenyang 110016, China; School of Materials Science and Engineering, University of Science and Technology of China, Shenyang 110016, China

**Keywords:** MoSi_2_N_4_, 2D layered materials, homologous compounds, iDPC-STEM, superconductivity

## Abstract

The number and stacking order of layers are two important degrees of freedom that can modulate the properties of 2D van der Waals (vdW) materials. However, the layers’ structures are essentially limited to the known layered 3D vdW materials. Recently, a new 2D vdW material, MoSi_2_N_4_, without known 3D counterparts, was synthesized by passivating the surface dangling bonds of non-layered 2D molybdenum nitride with elemental silicon, whose monolayer can be viewed as a monolayer MoN (-N-Mo-N-) sandwiched between two Si-N layers. This unique sandwich structure endows the MoSi_2_N_4_ monolayer with many fascinating properties and intriguing applications, and the surface-passivating growth method creates the possibility of tuning the layer's structure of 2D vdW materials. Here we synthesized a series of MoSi_2_N_4_(MoN)_4n_ structures confined in the matrix of multilayer MoSi_2_N_4_. These super-thick monolayers are the homologous compounds of MoSi_2_N_4_, which can be viewed as multilayer MoN (Mo_4n+1_N_4n+2_) sandwiched between two Si-N layers. First-principles calculations show that MoSi_2_N_4_(MoN)_4_ monolayers have much higher Young's modulus than MoN, which is attributed to the strong Si-N bonds on the surface. Importantly, different from the semiconducting nature of the MoSi_2_N_4_ monolayer, the MoSi_2_N_4_(MoN)_4_ monolayer is identified as a superconductor with a transition temperature of 9.02 K. The discovery of MoSi_2_N_4_(MoN)_4n_ structures not only expands the family of 2D materials but also brings a new degree of freedom to tailor the structure of 2D vdW materials, which may lead to unexpected novel properties and applications.

## Teaser

We discovered superconducting homologous compounds of 2D MoSi_2_N_4_, MoSi_2_N_4_(MoN)_4n_, which provides a new strategy to tailor the structure and properties of 2D materials by phase homology.

## INTRODUCTION

Van der Waals (vdW) layered materials have strong in-plane chemical bonds but weak interaction between adjacent layers, and therefore readily approach two-dimensional (2D) limits by diverse methods. This leads to many layer-dependent novel phenomena and unique properties that are absent in their three-dimensional (3D) bulk counterparts [1–14]. For example, monolayer graphene is a gapless semimetal, while bilayer graphene shows a gate-tunable bandgap or superconducting properties depending on the stacking order [1,6,15,16]; monolayer MoS_2_ is a direct bandgap semiconductor, whereas multilayer MoS_2_ is an indirect bandgap semiconductor [8]; monolayer CrI_3_ is an Ising ferromagnet, while bilayer CrI_3_ displays suppressed magnetization with a metamagnetic effect [17]. Therefore, the number and stacking of layers are two important degrees of freedom that can modulate the properties of vdW layered 2D materials. However, the layers’ structures are essentially limited to the known 3D layered vdW materials. Making a multiple-atomic-layer unit using vdW heterostructures of different 2D crystals creates a new possibility of tuning the layer properties of 2D materials. For example, MnBi_2_Te_4_/Bi_2_Te_3_ superlattices exhibit many exotic physical phenomena such as an intrinsic ferromagnetic topological state [18], large magnetic gap [19], quantum anomalous Hall regime [20] and controllable magnetic properties [21]. However, the unit in such a material as MnBi_2_Te_4_/Bi_2_Te_3_ is integrated by vdW interaction rather than the chemical bonding that is present in the layer of vdW materials.

Recently, we synthesized a new septuple-atomic-layer 2D vdW material without known 3D counterpart, MoSi_2_N_4_, by passivating the surface dangling bonds of non-layered 2D molybdenum nitride with elemental silicon in a chemical vapor deposition (CVD) process [22]. The MoSi_2_N_4_ monolayer can be viewed as a MoN_2_ layer sandwiched between two Si-N layers via chemical bonds, in which the MoN_2_ layer is a unit-cell-thick MoN slice (-N-Mo-N-) with N-terminating atomic layers on both sides, i.e. monolayer MoN. The structure of such a unique sandwiched layer means that monolayer MoSi_2_N_4_ is a very stable 2D semiconductor with potentially higher carrier mobility and better mechanical strength than monolayer MoS_2_. Furthermore, it is expected to have valley-contrasting properties, anomalous high thermal conductivity, outstanding optical properties, piezoelectricity, ferroelectricity, magnetic properties, efficient Ohmic contacts and photocatalytic and electrocatalytic activities with potential applications in valleytronics, nanoelectronics, optoelectronics and energy conversion [23–39]. Importantly, many such sandwich-structured 2D vdW materials with a general formula of MA_2_Z_4_ have been predicted [22,40,41], which will further expand the properties of 2D materials, and the surface-passivating CVD growth method creates the possibility of tailoring the layer structures of such 2D vdW materials.

Here, we synthesized a series of super-thick sandwich-structured vdW monolayers with a formula of MoSi_2_N_4_(MoN)_4n_ confined in the matrix of MoSi_2_N_4_ multilayer crystals by surface-passivating CVD. These super-thick monolayers are the homologous compounds of MoSi_2_N_4_, which can be viewed as multilayer MoN (Mo_4n+1_N_4n+2_) sandwiched between two Si-N layers. Among them, MoSi_2_N_4_(MoN)_4_ is predicted to be a phonon-mediated superconductor with a transition temperature of 9.02 K, and to have a higher Young's modulus than MoN. The discovery of MoSi_2_N_4_(MoN)_4n_ structures not only expands the family of 2D materials but also brings a new degree of freedom with which to tailor the structure of 2D vdW materials.

## RESULTS

MoSi_2_N_4_(MoN)_4n_ homogenous compounds were synthesized by the surface-passivating CVD, similar to the growth of MoSi_2_N_4_ [22]. A Cu/Mo bilayer was used as the substrate with the underlying Mo foil as Mo source, NH_3_ gas as the nitrogen source and a quartz plate as silicon source. The feeding rate of NH_3_ gas plays a key role in the structure of the products. At a low feeding rate of NH_3_ (3 sccm), strict monolayer MoSi_2_N_4_ film was obtained, whereas multilayer MoSi_2_N_4_ flakes were synthesized with increasing the flow rate of NH_3_. All the structures were characterized by recently developed integrated and differentiated differential phase contrast (iDPC and dDPC) scanning transmission electron microscopy (STEM) techniques [42–44]. The iDPC- and dDPC-STEM linearly correspond to the electrostatic potential field and charge density distribution field of a thin sample, respectively [42]. For high-angle annular dark-field (HAADF) STEM, it is challenging to image the light elements such as H, C, N, O in crystalline materials, especially for those containing heavy elements, since HAADF intensity is roughly proportional to *Z*^2^ (*Z*, atomic number) [45]. However, the iDPC intensity is roughly proportional to *Z*, thus giving rise to much stronger contrast enhancement of light elements among heavy elements [46].

**Figure 1.**
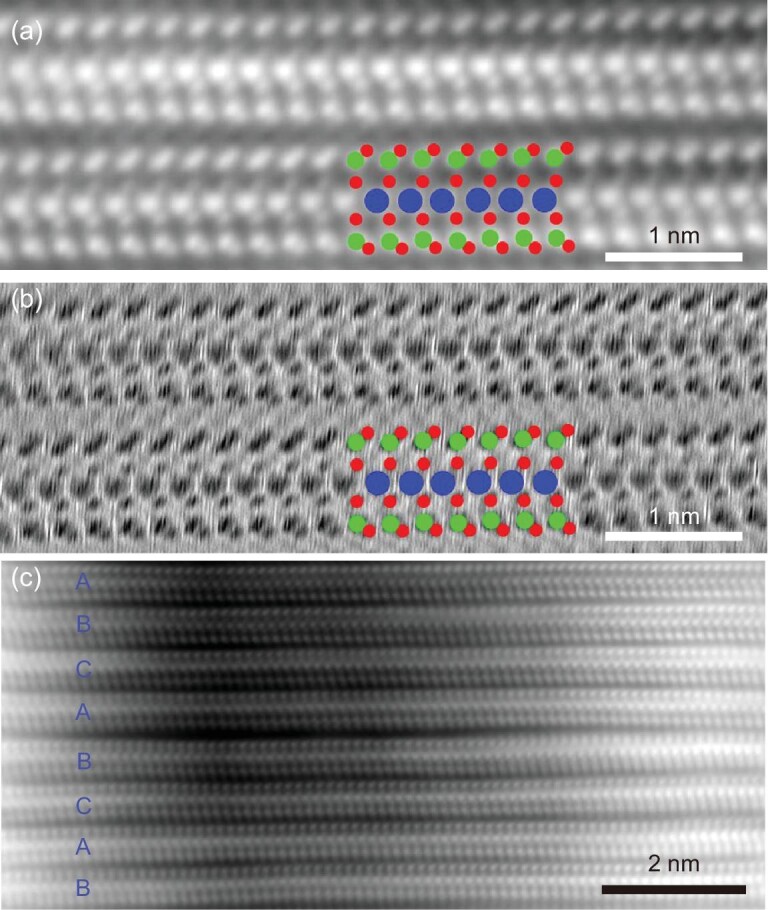
The atomic structure of multilayer MoSi_2_N_4_. (a and b) iDPC (a) and the corresponding dDPC (b) images of bilayer MoSi_2_N_4_, clearly showing the atomic sites of Mo (blue balls), Si (green balls) and N (red balls). (c) iDPC image of a rhombohedral-structured multilayer MoSi_2_N_4_ with ABC-typed stacking order.

We first characterized the crystalline structure of MoSi_2_N_4_; the N atomic occupation and layer stacking sequences had not been experimentally confirmed in our previous work [22]. Different from HAADF-STEM images, the cross-sectional iDPC- and dDPC-STEM images clearly show the exact sites of all the N atoms in MoSi_2_N_4_ besides Mo and Si, which form a coupling configuration of tetrahedra and triangular prisms around Si and Mo, respectively (Fig. 1a and b). This structure is completely consistent with our reported MoSi_2_N_4_ structure in which the N atomic occupation was finally confirmed by density functional theory (DFT) calculations [22].

The stacking order of MoSi_2_N_4_ layers determines the 3D crystallographic structure of MoSi_2_N_4_ bulk phase. From a geometric point of view, there are multiple stacking orders for MoSi_2_N_4_ layers including AA, AB, A${\mathrm{\bar{A}}}$, A${\mathrm{\bar{B}}}$, A${\mathrm{\bar{C}}}$ and ABC. The symbols of ${\mathrm{\bar{A}}}$, ${\mathrm{\bar{B}}}$, ${\mathrm{\bar{C}}}$ represent 180° rotation against A, B, C, respectively. Supplementary Table S1 shows the formation energies of different stacking orders (corresponding atomic models shown in Supplementary Fig. S1), in which the ABC-stacked MoSi_2_N_4_ phase is more energy-preferable. By checking dozens of samples, we found that ABC stacking shows the preference of long-range order compared with AA and AB stacking (Fig. 1c and Supplementary Fig. S2), while A${\mathrm{\bar{A}}}$, A${\mathrm{\bar{B}}}$, A${\mathrm{\bar{C}}}$ stacking orders were not observed. Therefore, the CVD-grown multilayer MoSi_2_N_4_ materials are predominantly rhombohedral-structured crystals with ABC stacking, whose crystallographic information is displayed in Supplementary Table S2.

**Figure 2.**
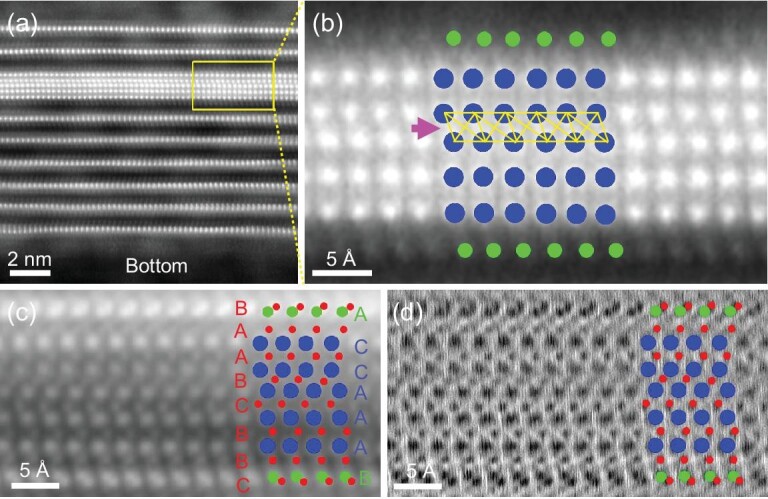
The atomic structure of MoSi_2_N_4_(MoN)_4_. (a and b) HAADF-STEM image of MoSi_2_N_4_(MoN)_4_ confined in multilayer MoSi_2_N_4_ (a) and the zoomed-in view of the MoSi_2_N_4_(MoN)_4_ (b). (c and d) iDPC (c) and dDPC (d) images of MoSi_2_N_4_(MoN)_4_. The blue, green and red balls represent the Mo, Si and N atoms, respectively.

Importantly, a thicker MoSi_2_N_4_(MoN)_4_ monolayer structure was formed in the matrix of multilayer MoSi_2_N_4_ by introducing more NH_3_. Large-field STEM images show that this structure looks like a MoSi_2_N_4_ intercalation compound (Supplementary Fig. S3), in which the intercalant contains Mo and N, confirmed by electron energy loss spectroscopy (EELS) (Supplementary Fig. S4). HAADF-STEM images show that it is composed of five Mo atomic layers sandwiched by two Si atomic layers (Fig. 2a and b). Further iDPC- and dDPC-STEM characterizations reveal eight N atomic layers interdigitating with Mo and Si layers, in which four internal N layers occupy the interstitial sites of triangular prisms and a peculiar layer of octahedra of Mo frame to form a MoN configuration, while the four external N layers form Si-N tetrahedra with the two Si atomic layers on two sides (Fig. 2c and d). These results suggest that the MoSi_2_N_4_(MoN)_4_ layer is built up by 15 atomic layers and can be viewed as a five-layer MoN (Mo_5_N_6_) sandwiched between two Si-N layers. In this structure, the two outer Mo atomic layers align with the multilayer MoSi_2_N_4_ matrix. The spatial confinement effect of the MoSi_2_N_4_ matrix results in the formation of a Mo octahedron stacking fault layer (Fig. 2b), which reduces the volume expansion of MoSi_2_N_4_(MoN)_4_ in the MoSi_2_N_4_ matrix (Supplementary Fig. S5).

Phase homology refers to a series of compounds built on the same structural principle with certain modules expanding in various dimensions by regular increments [47,48]. Thus, MoSi_2_N_4_(MoN)_4_ is a homologous compound of MoSi_2_N_4_ based on the structural expansion of internal MoN configuration. Notably, MoSi_2_N_4_(MoN)_4_ is different from the intercalated compounds, in which guest species such as atoms, ions and molecules occupy the interlayer space of vdW layered crystals without changing the layers’ structures [49–52]. It is also different from the recently reported covalently bonded 2D layered materials engineered by self-intercalation, where the intercalated atomic layers covalently bond to pristine 2D materials [49].

**Figure 3.**
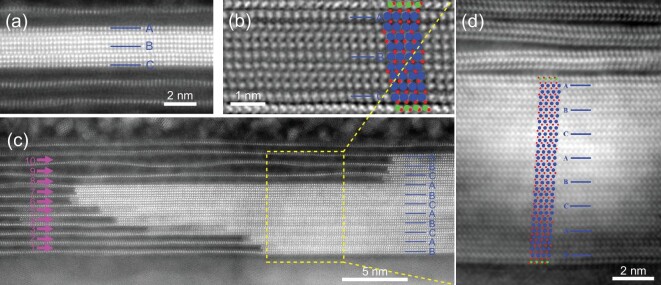
The atomic structure of super-thick MoSi_2_N_4_(MoN)_4n_. (a and b) HAADF-STEM image of MoSi_2_N_4_(MoN)_8_ confined in multilayer MoSi_2_N_4_ (a) and iDPC image of MoSi_2_N_4_(MoN)_8_ (b). The A, B and C lines indicate the Mo atomic layers aligned with the MoSi_2_N_4_ matrix. (c and d) HAADF-STEM image of one end of the MoSi_2_N_4_(MoN)_4n_ confined in 11-layer MoSi_2_N_4_ (c) and iDPC image of MoSi_2_N_4_(MoN)_28_ (d). The blue, green and red balls represent the Mo, Si and N atoms, respectively.

Interestingly, much thicker homologous MoSi_2_N_4_(MoN)_4n_ monolayers with *n* = 2, 3, 4, 5, 6, 7, 8, 9, 10 can also be synthesized (Fig. 3 and Supplementary Fig. S6). Figure 3a and b show the HAADF and iDPC images of MoSi_2_N_4_(MoN)_8_, respectively. Note that the Mo atomic layers aligned with the MoSi_2_N_4_ matrix maintain stable ABC stacking, the same as the pure MoSi_2_N_4_ multilayers, suggesting that they inherited the structure of the matrix. Like MoSi_2_N_4_(MoN)_4_, the two outermost Si-N layers of MoSi_2_N_4_(MoN)_8_ are preserved, while the others are replaced with Mo-N layers. The MoSi_2_N_4_(MoN)_8_ layer is built up by 23 atomic layers and can be viewed as a nine-layer MoN (Mo_9_N_10_) sandwiched between two Si-N layers (Fig. 3b). Figure 3c exhibits the HAADF image of a super-thick MoSi_2_N_4_(MoN)_40_ containing 87 atomic layers, which is the thickest vdW monolayer structure reported so far. As shown in the iDPC image (Fig. 3d), all the MoN configurations are similar in between the Mo atomic layers aligned with the MoSi_2_N_4_ matrix, forming a consecutive MoN phase (Supplementary Fig. S7). However, we have also observed MoSi_2_N_4_(MoN)_4n_ isomers with different N arrangements on the surface and/or mirror-symmetric Mo-N inner layers (Supplementary Fig. S8). In principle, the thickness of the homologous compound MoSi_2_N_4_(MoN)_4n_ monolayer can be further increased.

**Figure 4.**
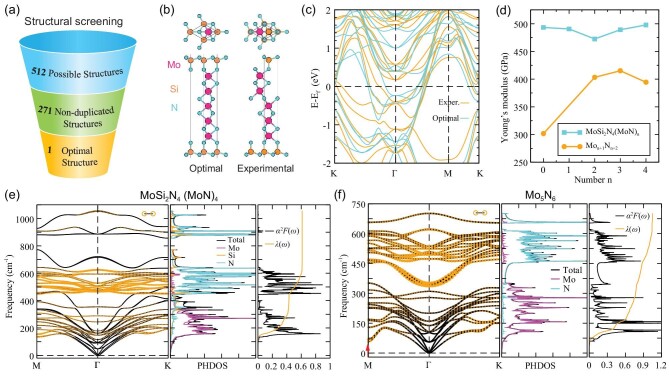
Calculated structures and properties of MoSi_2_N_4_(MoN)_n_. (a) Diagram illustrating the process of structure screening of MoSi_2_N_4_(MoN)_4_. (b and c) The atomic models (b) and electronic band structures (c) of the energetically favorable structure and experimental structure of MoSi_2_N_4_(MoN)_4_. (d) Young's modulus of MoSi_2_N_4_(MoN)_n_ and Mo_n+1_N_n+2_ for n = 0−4. The Young's modulus of Mo_2_N_3_ was not given due to its mechanical instability. (e and f) Phonon dispersions, vibrational PHDOS, Eliashberg function and accumulated EPC constants of MoSi_2_N_4_(MoN)_4_ (e) and Mo_5_N_6_ (f), where the circles in phonon dispersion plots represent the phonon linewidth *γ***_q_***_ν_*, related to the contribution to *α*^2^*F*(*ω*) at each **q** and each mode *ν*.

The formation of MoSi_2_N_4_(MoN)_4n_ in the matrix of MoSi_2_N_4_ shows the potential for tailoring the layer structures of 2D vdW materials by changing the number of sandwiched MoN layers. It is reasonable to expect that isolated MoSi_2_N_4_(MoN)_n_ could be synthesized by precisely designing the growth process and parameters. We used first-principles DFT calculations to evaluate the structure and properties of the MoSi_2_N_4_(MoN)_n_ with n = 1−4. By changing the positions of Mo and N atoms based on the symmetry analyses and structural screening, energetically favorable atomic structures were obtained for each MoSi_2_N_4_(MoN)_n_, as shown in Supplementary Fig. S9; these are dynamically stable without any imaginary frequencies found in the phonon dispersions (Supplementary Fig. S10). The calculated energetically favorable structure of isolated MoSi_2_N_4_(MoN)_4_ screened from 512 candidates contains only MoN triangular prisms (Fig. 4a and b), which are slightly different from the experimentally observed ones confined in bilayer MoSi_2_N_4_ (Fig. 2c and d). The energy of the calculated MoSi_2_N_4_(MoN)_4_ structure is 0.108 eV/atom lower than that of the experimental structure. The presence of the stacking fault layer leads to a small difference in the electronic band structures, but both show metallic characteristics (Fig. 4c). To identify the uniqueness of these homologous compounds, we studied the energetically favorable isolated MoSi_2_N_4_(MoN)_n_ via a comparison with the corresponding sandwiched Mo_n+1_N_n+2_ (Supplementary Fig. S11).

Figure 4d and Supplementary Table S3 show that MoSi_2_N_4_(MoN)_n_ (n = 0−4) has much higher Young's modulus than the sandwiched Mo_n+1_N_n+2_. The Young's modulus of Mo_n+1_N_n+2_ strongly depends on the number of MoN layers, which approaches that of bulk MoN crystals (431.32 GPa) at four layers. In contrast, the Young's modulus of MoSi_2_N_4_(MoN)_n_ only shows slight changes with the number of MoN layers. These results suggest the significant contribution of the outmost Si-N layers to the excellent mechanical properties of MoSi_2_N_4_(MoN)_n_. To understand the physical origin, we analyzed the bonding characteristics of MoSi_2_N_4_(MoN)_n_ and Mo_n+1_N_n+2_ compounds by deriving the electron localization function (ELF). ELF = 1 and ELF = 0 mean perfect electron localization and delocalization, respectively, while ELF = 0.5 corresponds to the electron-gas-like pair probability [53]. As shown in Supplementary Fig. S12, the electrons are largely localized around N atoms, which indicates the ionic bond characteristics of Mo-N bonds and Si-N bonds in the MoSi_2_N_4_(MoN)_n_ and Mo_n+1_N_n+2_ (n = 0−4). Compared to Mo atoms, the almost-zero ELF value around Si atoms suggests no valence electrons around Si atoms, indicating that Si-N bonds are much stronger than Mo-N bonds. Therefore, the surface Si-N layers are responsible for the superior Young's modulus of MoSi_2_N_4_(MoN)_n_.

More importantly, different from the semiconducting characteristic of monolayer MoSi_2_N_4_, MoSi_2_N_4_(MoN)_n_ (n = 1−4) monolayers were identified as phonon-mediated superconductors, the superconductivity of which originates from the formation of electron Cooper pairs induced by the interaction between electrons and phonons [54,55]. To illustrate this point, we further investigated the electron-phonon coupling (EPC) and superconductivity in MoSi_2_N_4_(MoN)_n_ (n = 1−4). The phonon dispersions, vibrational phonon density of states (PHDOS), Eliashberg function and accumulated EPC constants of MoSi_2_N_4_(MoN)_n_ are given in Fig. 4e, Supplementary Fig. S13 and Supplementary Table S4. It is worth noting that the superconducting transition temperature (*T*_c_) and EPC constants (*λ*) of MoSi_2_N_4_(MoN)_n_ increase with the increase in the number of MoN layers for n = 1−3 (Supplementary Table S4). Interestingly, the *T*_c_ and *λ* of MoSi_2_N_4_(MoN)_3_ are higher than those of MoSi_2_N_4_(MoN)_4_. We found that this is mainly caused by the softening of the acoustic mode of MoSi_2_N_4_(MoN)_3_, as marked by the arrow in Supplementary Fig. S13c. To investigate the influence of Si-N layers on the properties of MoSi_2_N_4_(MoN)_4_, the *T*_c_ and *λ* of Mo_5_N_6_ were derived from its Eliashberg function and accumulated EPC constants (Fig. 4f). We found that the highest phonon frequency is 701.27 cm^−1^ for Mo_5_N_6_, lower than that of MoSi_2_N_4_(MoN)_4_ (1051.24 cm^−1^). This indicates a weaker bonding interaction in Mo_5_N_6_, which agrees well with the results of Young's modulus calculations. Furthermore, the *λ* value of Mo_5_N_6_ is up to 1.05, which is mainly contributed by the Mo-related vibrational modes at frequencies lower than 350 cm^−1^, and partially originated from N-related vibrational modes at frequencies higher than 350 cm^−1^. MoSi_2_N_4_(MoN)_4_ shows a similar feature with Mo_5_N_6_ at frequencies from 0 to 700 cm^−1^. The difference is that the softening of the acoustic mode of Mo_5_N_6_ (marked by a arrow in Fig. 4f) significantly enhances its *λ*, and four parabolic optical branches contribute to a large *α*^2^*F*(*ω*) value at N-related frequencies from 350 to 450 cm^−1^. We also checked the local density of states (LDOS) of Mo_5_N_6_, and the band structure and partial density of states (PDOS) of MoSi_2_N_4_(MoN)_n_ and Mo_n+1_N_n+2_ (n = 0−4) (Supplementary Figs S14–S16). It was found that, (i) the outmost N atoms mainly contribute to the LDOS of N atoms of Mo_5_N_6_ near the Fermi level; (ii) compared to Mo_n+1_N_n+2_, MoSi_2_N_4_(MoN)_n_ shows a different band structure with nearly absent *p*-orbital components of N atoms near the Fermi level in the PDOS, demonstrating that the Si-N layers significantly restructure the electronic structure of MoSi_2_N_4_(MoN)_n_. Such reconstruction can also be explained by the charge density difference (CDD) of MoSi_2_N_4_(MoN)_4_ (Supplementary Fig. S17). Therefore, the N atoms of MoSi_2_N_4_(MoN)_4_ do not show an apparent contribution to its *λ* and *α*^2^*F*(*ω*) when compared with their phonon linewidth (the size of the circle in phonon dispersions) at frequencies from 350 to 450 cm^−1^ (Fig. 4e and f). As a result, the Si-N layers change the *T*_c_ from 19.74 K (Mo_5_N_6_) to 9.02 K (MoSi_2_N_4_(MoN)_4_).

## DISCUSSION

MoSi_2_N_4_(MoN)_n_ represents a new state of matter, the 2D homologous compound, which unlocks the engineering of monolayer 2D vdW materials by expanding the sandwiched building blocks. Besides MoSi_2_N_4_, recently predicted septuple-atomic-layer MA_2_Z_4_ family materials [22,40] also have the potential to form MA_2_Z_4_(MZ)_n_ homologous compounds based on their similar sandwich structure to MoSi_2_N_4_. These homologous compounds may lead to many unexpected novel properties and applications that could not be achieved with the existing 2D vdW materials. The surface-passivating CVD strategy has the potential to synthesize such materials and homologous compounds of other sandwich structured 2D vdW materials, such as MnBi_2_Te_4_ [56], which can be viewed as MnTe passivated with Bi_2_Te_3_. However, it is still challenging to controllably achieve isolated MA_2_Z_4_(MZ)_n_.

## METHODS

### CVD growth of multilayer MoSi_2_N_4_ and MoSi_2_N_4_(MoN)_4n_

Multilayer MoSi_2_N_4_ and MoSi_2_N_4_(MoN)_4n_ were grown by the CVD method that was reported previously for the growth of monolayer MoSi_2_N_4_ [22]. The differences were the flow rate of NH_3_, which was 3, 6−8 and ≥10 sccm for the growth of the monolayer, multilayer MoSi_2_N_4_ and MoSi_2_N_4_(MoN)_4n_, respectively. A NH_3_ feeding rate of 10 sccm and a growth time of 1 hour are the fundamental parameters to synthesize MoSi_2_N_4_(MoN)_4_. On this basis, increasing the values of either parameter would promote the increase in thickness of MoSi_2_N_4_(MoN)_4n_.

### Structural characterizations

HAADF, iDPC, dDPC and EELS measurements were performed in STEM mode on an FEI Titan Cubed Themis G2 300 instrument equipped with a high-brightness field-emission gun (X-FEG), double spherical aberration corrector and a monochromator. Detailed STEM imaging parameters were given as follows: crystalline orientation of cross-sectional MoSi_2_N_4_ samples ([2 110]), typical camera length (115 mm for HAADF and 285 mm for iDPC and dDPC), collection semi-angle (47–200 mrad for HAADF and 5–27 mrad for iDPC and dDPC) and beam current (47 pA for HAADF and 25 pA for iDPC and dDPC). The cross-sectional TEM samples were fabricated by Tescan LYRA 3 XMU focused ion beam (FIB) microscope. To do that, the samples were transferred onto SiO_2_/Si substrate and deposited with a thin layer of Pt as a protection layer. The average background subtraction filter (ABSF), Wiener filter and high-pass filter were used to improve the signal-to-noise ratio and visibility of atomic-scale structures. The iDPC contrast enhancement of the outmost Si-N layers (Figs 2c, 3b and S8) possibly originates from the probe defocus effect and/or mass-thickness contrast of the thick sample containing heavy Mo element [57,58].

### Theoretical calculations

First-principles calculations were employed using the Vienna *ab initio* simulation package (VASP) [59,60] and Quantum ESPRESSO (QE) package [61,62]. In detail, the structural screening, electronic structures, ELF and Young's modulus were calculated by VASP. EPC constants and superconductivity were evaluated by QE. The details are described in the supplementary data.

## Supplementary Material

nwac273_Supplemental_File
