# Supplementary material for: Two-dimensional superconducting MoSi2N4(MoN)4n homologous compounds
Source: Natl Sci Rev. 2022 Nov 28;10(4):nwac273. doi: 10.1093/nsr/nwac273 (PMC11299712; doi:10.1093/nsr/nwac273)
Supplement: nwac273_Supplemental_File [file nwac273_supplemental_file.pdf]

Supplementary Information

Two-dimensional superconducting $MoSi_2N_4(MoN)_{4n}$ homologous compounds

Zhibo Liu[1,†], Lei Wang[1,2,†], Yi-Lun Hong[1,2,†], Xing-Qiu Chen[1,2], Hui-Ming Cheng[1,2,3] and Wencai Ren[1,2,*]

[1]Shenyang National Laboratory for Materials Science, Institute of Metal Research, Chinese Academy of Sciences, Shenyang 110016, China;

[2]School of Materials Science and Engineering, University of Science and Technology of China, Shenyang 110016, China;

[3]Shenzhen Institute of Advanced Technology, Chinese Academy of Sciences, Shenzhen 518055, China

* **Corresponding author.** E-mail: wcren@imr.ac.cn

† Equally contributed to this work.

For the VASP calculations, the exchange-correlation potential was adopted by the generalized gradient approximation (GGA) [1] of Perdew-Burke-Ernzerhof (PBE). The cutoff energy for plane-wave expansion was 500 eV and the k-point sampling grid in the relaxation and self-consistent step was 15 × 15 × 1 for monolayers, 15 × 15 × 5 and 15 × 15 × 3 for the stacking order calculations of bulk phase with two and three monolayers, respectively. And the optPBE-vdW was used to consider the vdW interactions between monolayers. The crystal structures were relaxed enough until the forces on each atom became less than 0.001 eV/Å and the energy difference on the primitive cell between the last two steps became less than 10^{-6} eV. A vacuum of 20 Å between layers with periodic images was considered to minimize the interactions between them. The Young's modulus of 2D materials [2] was calculated by (C11 × C22 - $C12^2$)/C11 × (*d* +3.1)/*D*, where *d* was the thickness of $MoSi_2N_4(MoN)_n$ and $Mo_{n+1}N_{n+2}$ monolayer (listed in Table S3), 3.1 was the assumed distance of 3.1 Å between two monolayers obtained from two times the van der Waals radius of the N atom (1.55 Å), and the *D* was the length of unit-cell along z-direction. For the QE calculations, the exchange-correlation potential was treated by local density approximation (LDA) [3] with norm-conserving pseudopotentials. The kinetic energy cutoff and the charge density cutoff of plane-wave basis were chosen to be 80 and 800 Ry. All the structures were fully relaxed to their equilibrium state such that the forces acting on each atom became smaller than 10^{-6} Ry/Bohr. Marzari-Vanderbilt cold smearing of 0.02 Ry was used to improve convergence. The self-consistent electron density was evaluated by employing a *k*-mesh of 24 × 24 × 1. The EPC and

superconductivity for each $MoSi_2N_4(MoN)_n$ (n = 1 – 4) and Mo_5N_6 were calculated by using the density functional perturbation theory (DFPT) [4], in which the q-mesh of 4 × 4 × 1 was used. Finally, the superconducting transition temperature T_c was derived by following Allen-Dynes-modified McMillan formula [5,6]

$$T_c = \frac{\omega_{\log}}{1.2}\exp[\frac{-1.04(1+\lambda)}{\lambda-\mu^*(1+0.62\lambda)}],$$

where λ is the electron-phonon coupling constant obtained by $\lambda(\omega_{max})$, μ^* is the effectively screened Coulomb interaction treated as a constant of 0.1, and $\omega_{\log}$ is the logarithmic average phonon frequencies. Both λ and $\omega_{\log}$ can be calculated from the Eliashberg function $\alpha^2F(\omega)$.

$$\lambda(\omega) = 2\int d\omega\, \alpha^2F(\omega)/\omega,$$

$$\omega_{\log} = \exp[\frac{2}{\lambda}\int_0^\infty \frac{d\omega}{\omega}\alpha^2F(\omega)\log\omega],$$

$$\alpha^2F(\omega) = \frac{1}{2\pi N(\mathrm{E_F})}\sum_{\mathbf{q}\nu}\delta(\omega-\omega_{\mathbf{q}\nu})\frac{\gamma_{\mathbf{q}\nu}}{\hbar\omega_{\mathbf{q}\nu}},$$

where ω is the frequencies of phonon, $\omega_{\mathbf{q}\nu}$ is the frequency of phonon of the mode ν at the wave vector **q**, and $\gamma_{\mathbf{q}\nu}$ is the phonon linewidth of the mode ν at the wave vector **q**, obtained from electron-phonon matrix element calculations performed in QE, indicating the interaction strength between electron and phonon. $N(\mathrm{E_F})$ is the density of states at Fermi level $\mathrm{E_F}$.

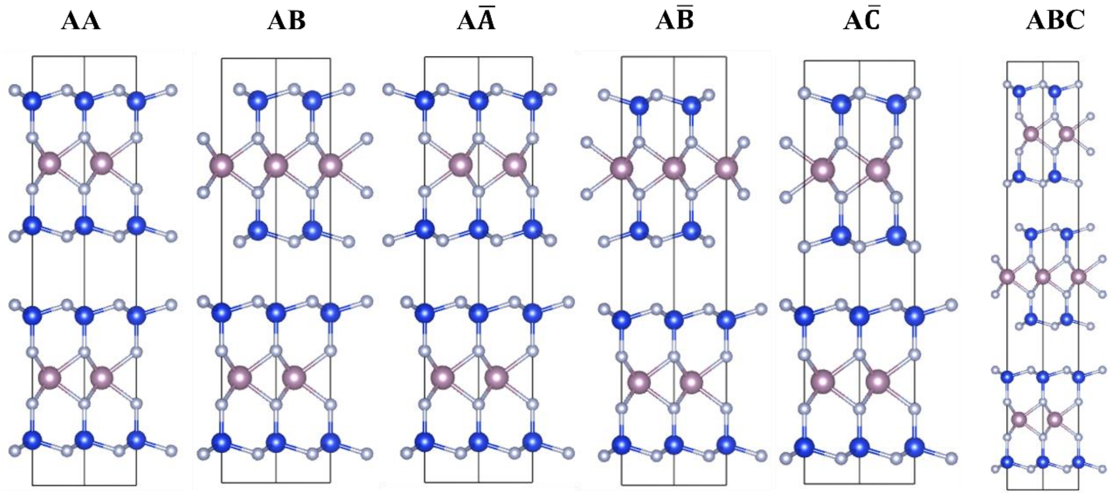

Figure S1. Stacking configurations of $MoSi_2N_4$.

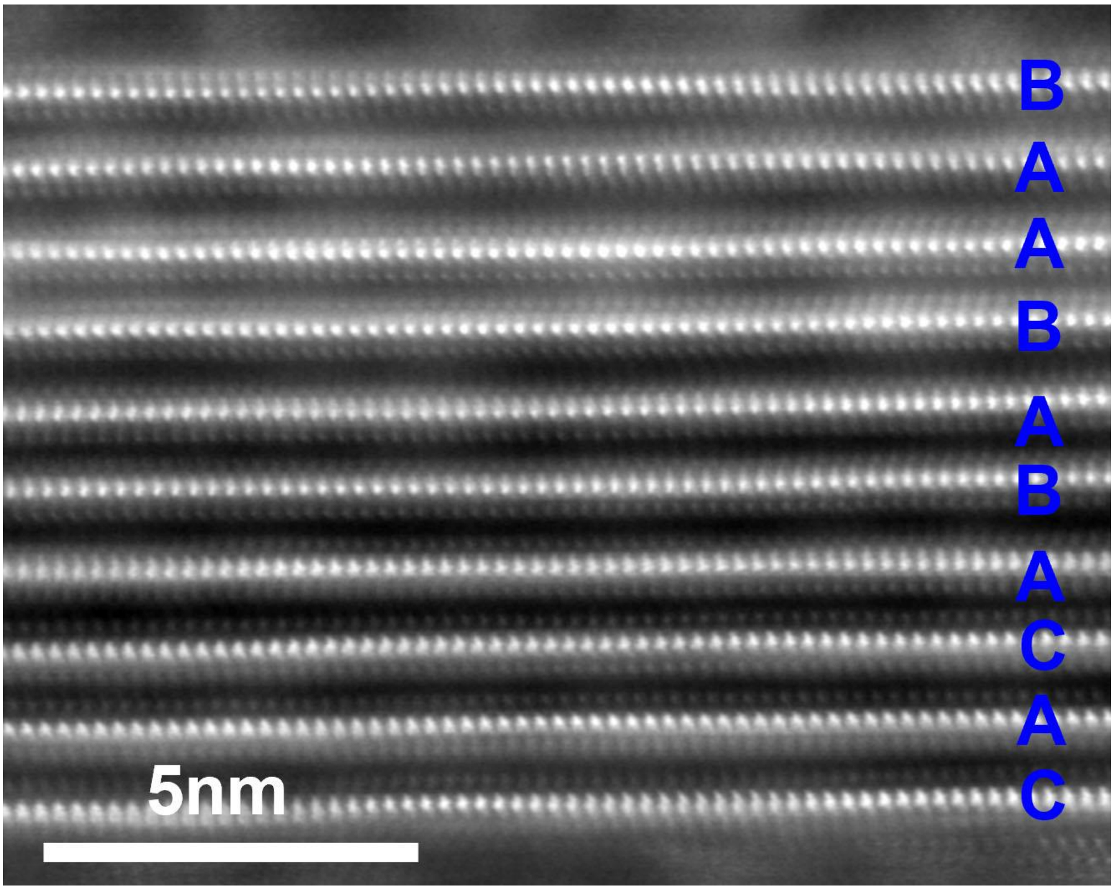

Figure S2. The multilayer $MoSi_2N_4$ structure containing AA, AB and AC stacking configurations.

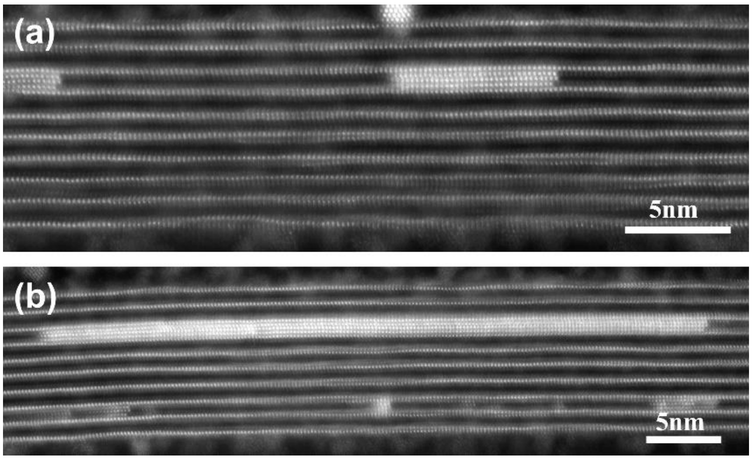

Figure S3. HAADF-STEM images of $MoSi_2N_4(MoN)_4$ confined in multilayer $MoSi_2N_4$.

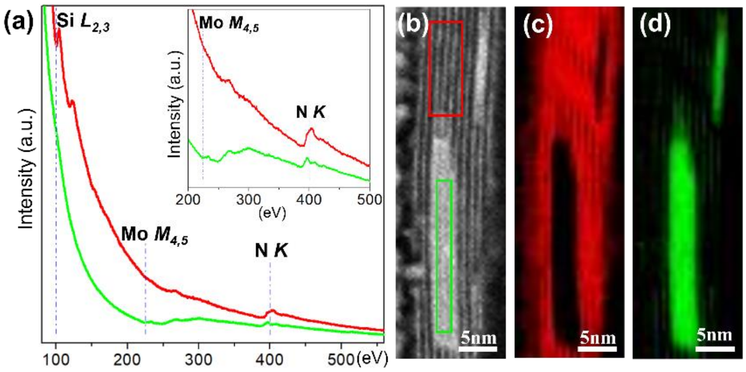

Figure S4. (a) EELS profiles of multilayer $MoSi_2N_4$ from the red frame and MoN from the green frame in (b), respectively. The red EELS profile contains the ionization edges of Si, Mo and N, while the green EELS profile only contains the ionization edges of Mo and N. (b) The HAADF-STEM image of multilayer $MoSi_2N_4$ with confined $MoSi_2N_4(MoN)_{4n}$. (c and d) The EELS mappings of $MoSi_2N_4$ area (c) derived from the red EELS profile in (a) and MoN area (d) derived from the green EELS profile in (a) based on the multivariate linear least square method.

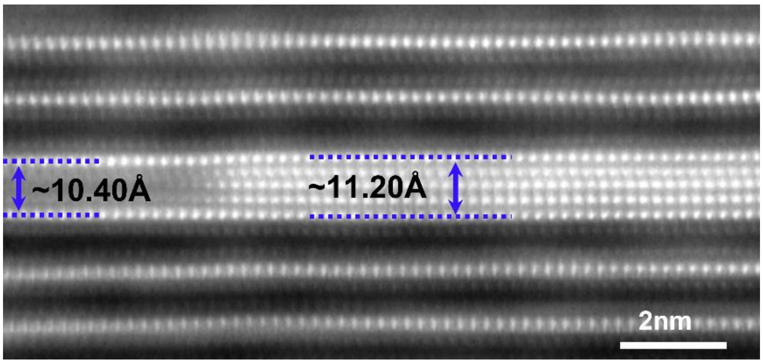

Figure S5. HAADF-STEM image of $MoSi_2N_4(MoN)_4$ confined in multilayer $MoSi_2N_4$. The thickness of $MoSi_2N_4(MoN)_4$ is slightly larger than that of bilayer $MoSi_2N_4$.

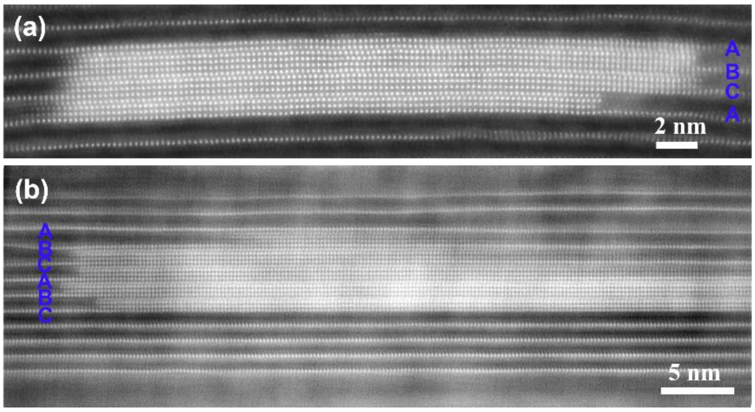

Figure S6. The HAADF-STEM images of $MoSi_2N_4(MoN)_{4n}$ confined in multilayer $MoSi_2N_4$ with n up to 3 (a) and 5 (b), respectively.

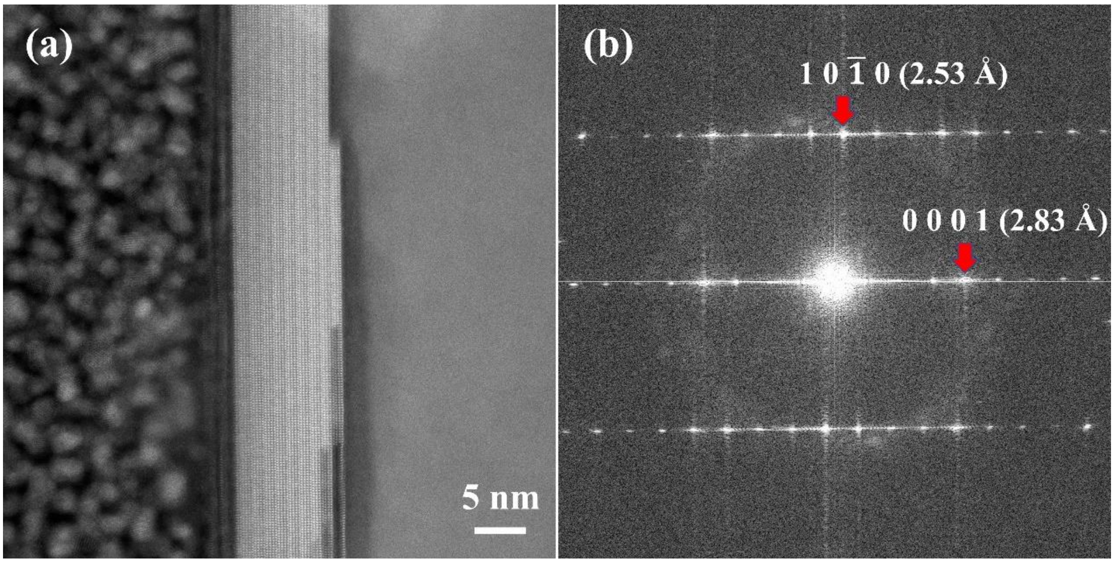

Figure S7. HAADF image of $MoSi_2N_4(MoN)_{36}$ (a) and the corresponding Fourier transformation (b). The obtained interplanar spacing corresponding to (1 0 $\bar{1}$ 0) and (0 0 0 1) index of MoN is 2.53 Å and 2.83 Å, respectively. Therefore, the lattice parameters of MoN corresponding to the inner Mo-N layers of $MoSi_2N_4(MoN)_{36}$ are a = b = 2.92 Å, c= 2.83 Å, which are consistent with the reported values of MoN [7].

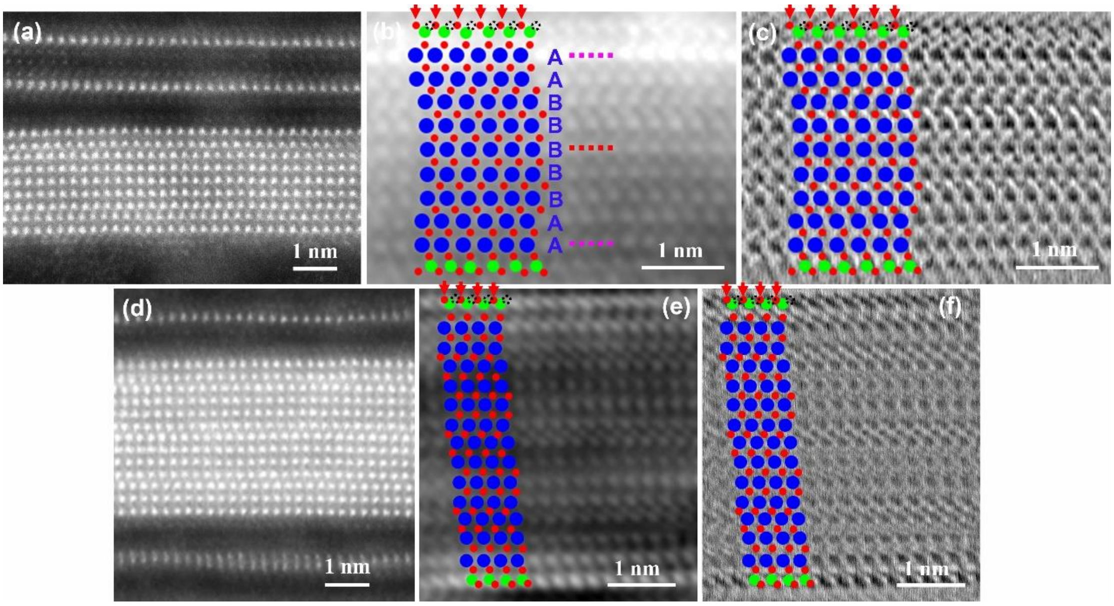

Figure S8. Abnormal configurations of $MoSi_2N_4(MoN)_{4n}$. (a-c) HAADF (a), iDPC (b) and dDPC (c) images of $MoSi_2N_4(MoN)_8$, which displays a mirror-symmetric Mo-N configuration with the abnormal N layer indicated by arrows. (d-f) HAADF (d), iDPC (e) and dDPC (f) images of $MoSi_2N_4(MoN)_{12}$, which displays an abnormal N layer indicated by arrows.

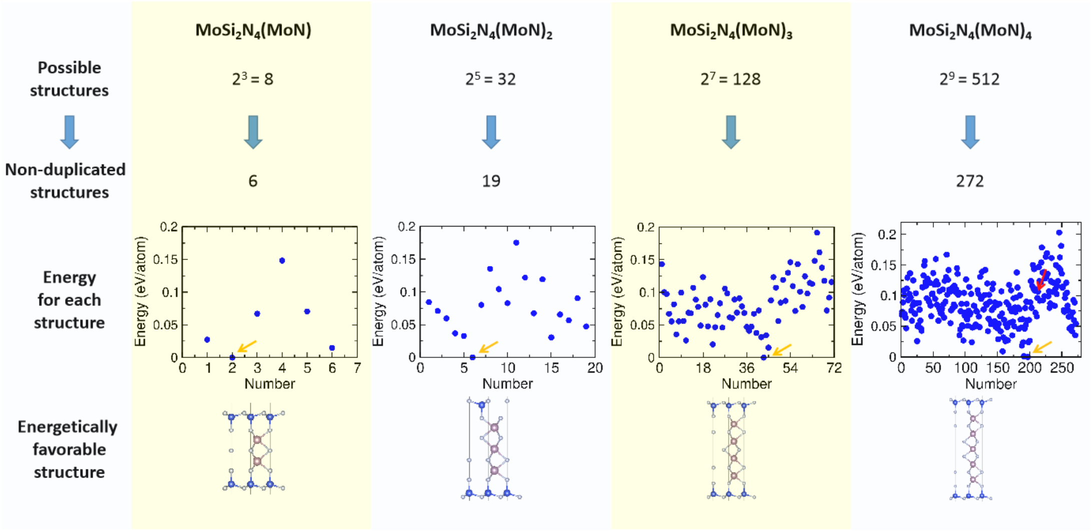

Figure S9. The screening of energetically favorable structure of $MoSi_2N_4(MoN)_n$ with n = 1 – 4. The orange arrows point to the energetically favorable structures of $MoSi_2N_4(MoN)_n$, and the red arrow points to the experimental lattice structure of $MoSi_2N_4(MoN)_4$.

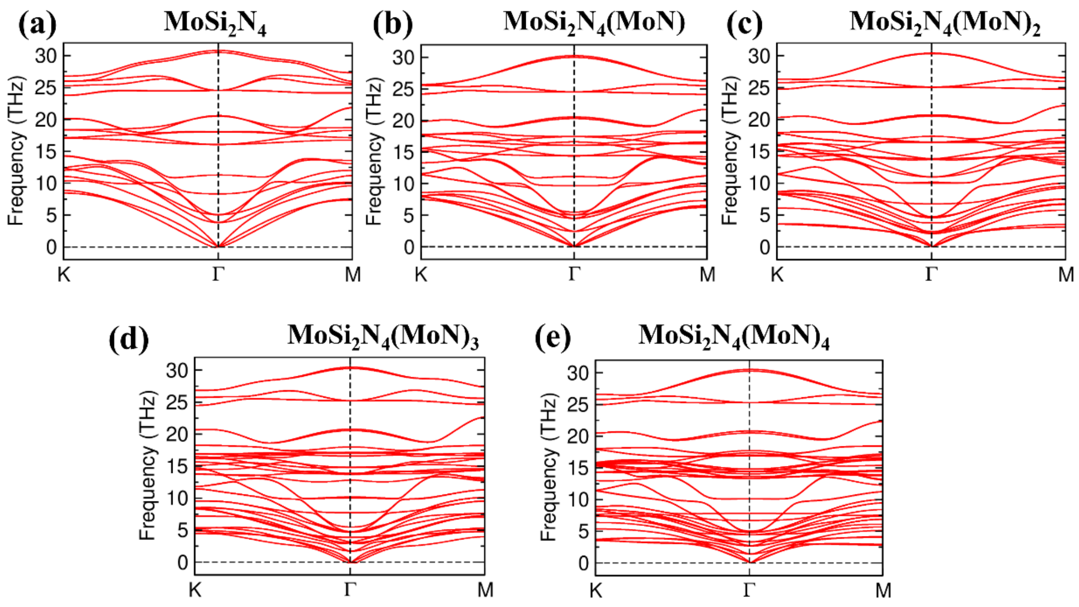

Figure S10. Phonon spectra of $MoSi_2N_4(MoN)_n$ (n = 0 – 4).

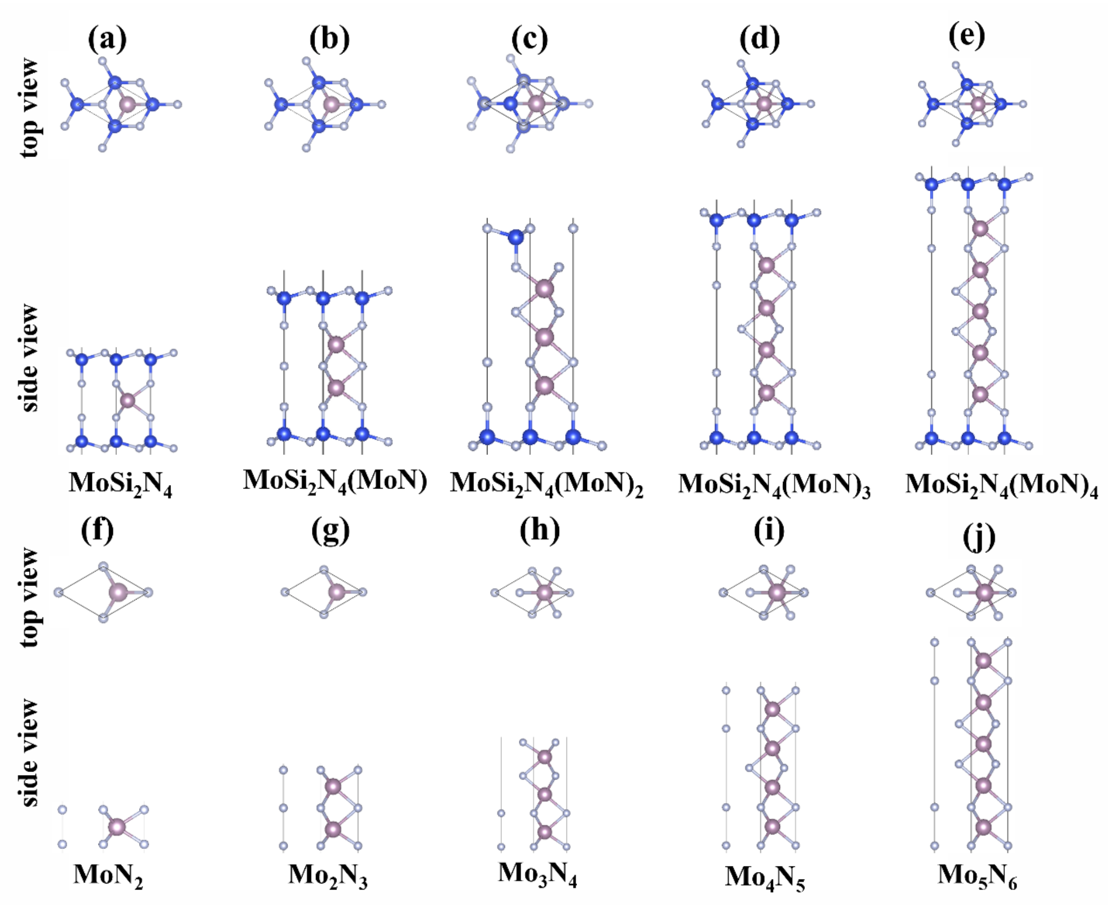

Figure S11. The lattice structures of $MoSi_2N_4(MoN)_n$ and $Mo_{n+1}N_{n+2}$ (n = 0 – 4). (a-e) The top view and side view of energetically favorable $MoSi_2N_4(MoN)_n$. (f-j) The top view and side view of $Mo_{n+1}N_{n+2}$, which was obtained by removing the top and bottom Si-N layers from $MoSi_2N_4(MoN)_n$.

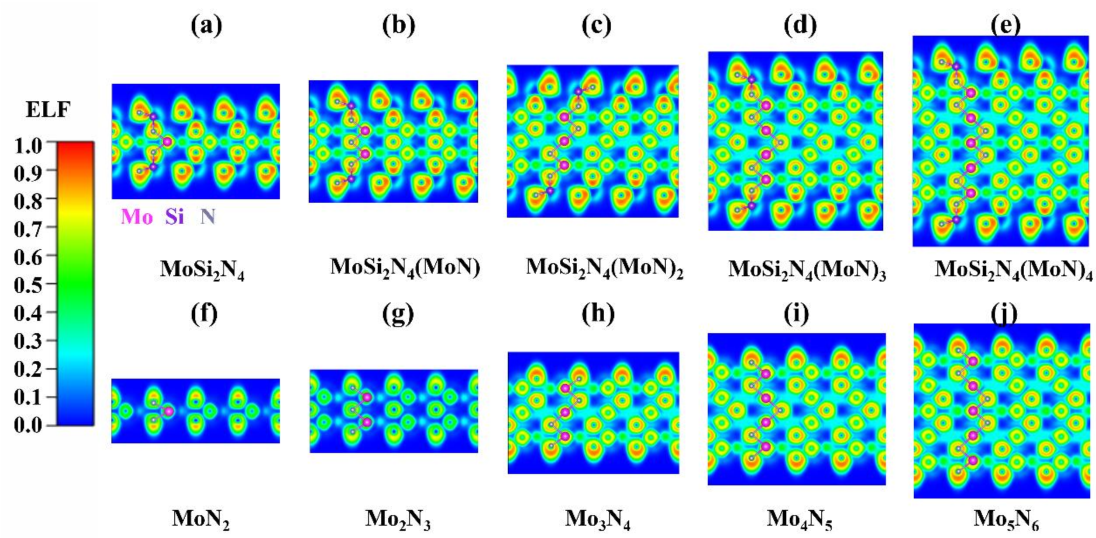

Figure S12. ELF of $MoSi_2N_4(MoN)_n$ and $Mo_{n+1}N_{n+2}$ (n = 0 – 4). The atoms and bonds are shown in each image.

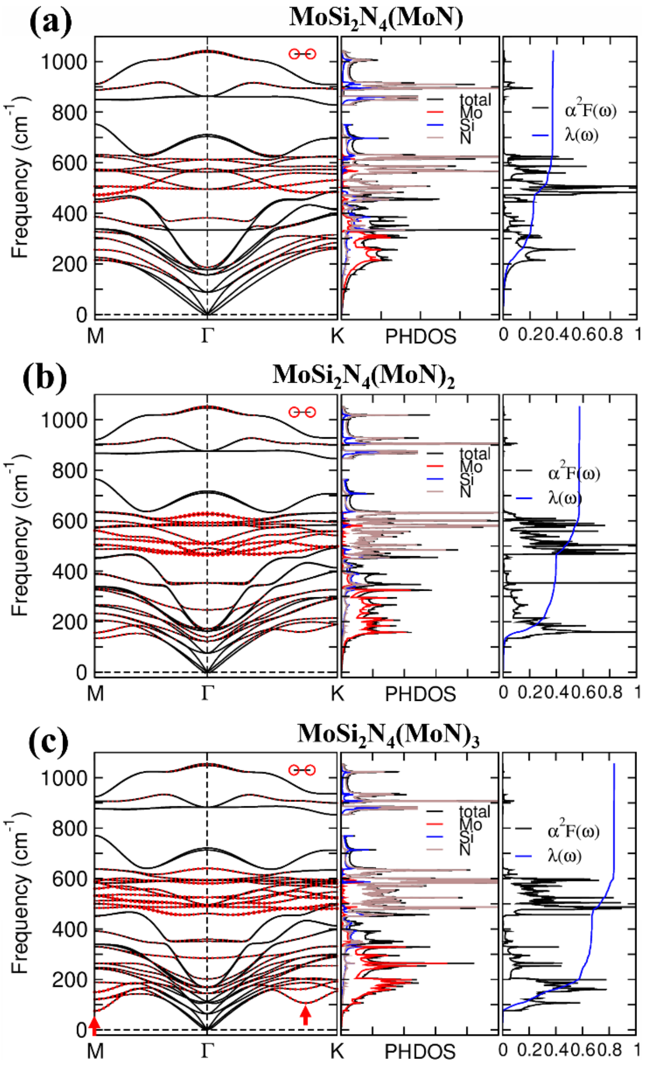

Figure S13. Phonon dispersions, PHDOS, Eliashberg function $\alpha^2F(\omega)$ and the electron–phonon coupling strength $\lambda(\omega)$ of $MoSi_2N_4(MoN)_n$ (n = 1 – 3). The area of the circles in the phonon dispersions represents the strength of phonon linewidth $\gamma_{\mathbf{q}\nu}$.

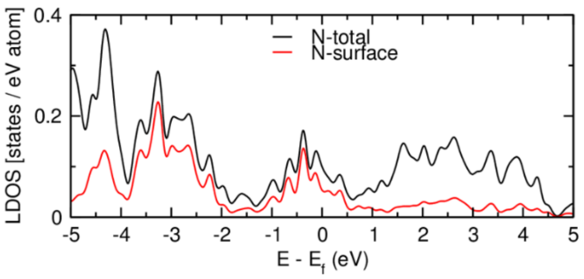

Figure S14. The LDOS of N atoms of Mo_5N_6. The black solid line denotes the LDOS of total N atoms of Mo_5N_6, and the red solid line denotes the LDOS of outmost N atoms of Mo_5N_6 on the surface. The results indicate that N atoms on the surface mainly contribute to the LDOS of the N atoms of Mo_5N_6 near Fermi level.

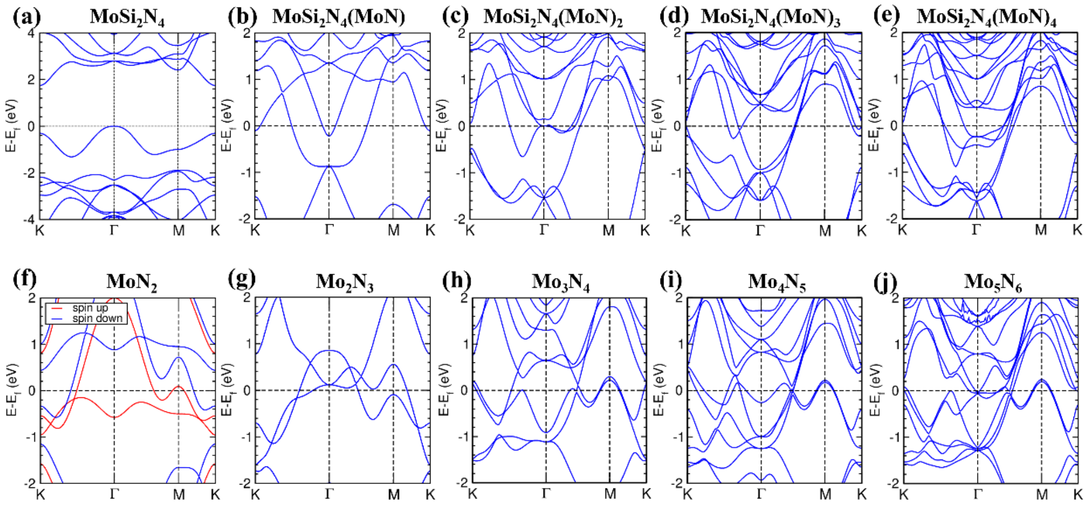

Figure S15. Band structures of $MoSi_2N_4(MoN)_n$ and $Mo_{n+1}N_{n+2}$ (n = 0 – 4).

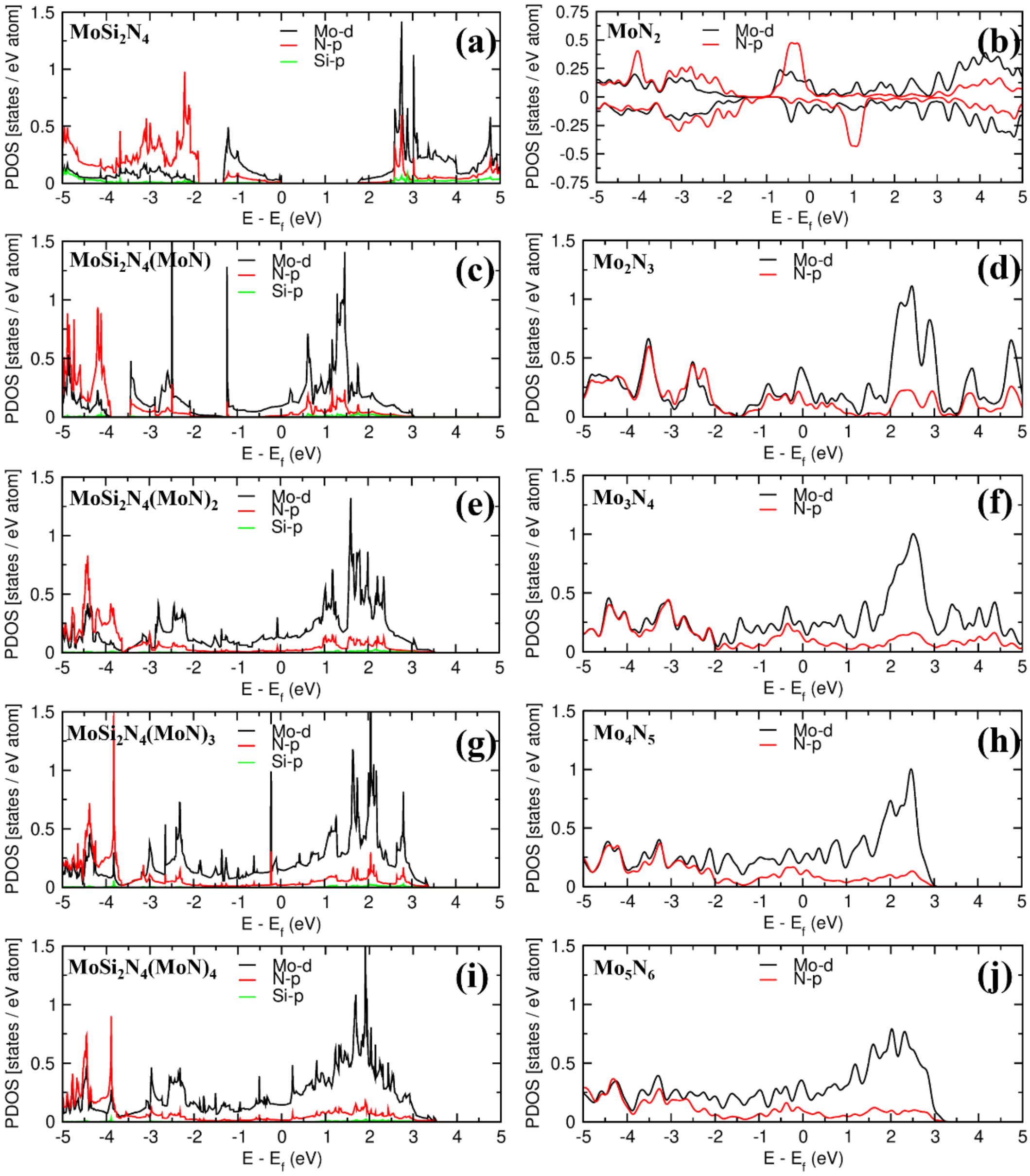

Figure S16. PDOS of $MoSi_2N_4(MoN)_n$ and $Mo_{n+1}N_{n+2}$ (n = 0 – 4). Compared with $Mo_{n+1}N_{n+2}$, the nearly absent *p*-orbital components of N atoms near the Fermi level indicate the reconstruction of electronic structure of $MoSi_2N_4(MoN)_n$ due to the addition of Si-N layers.

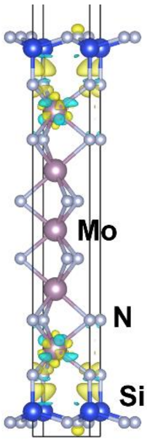

Figure S17. The charge density difference (CDD) of $MoSi_2N_4(MoN)_4$. The CDD was calculated by the formula of $\Delta\rho(r) = \rho(r)\{MoSi_2N_4(MoN)_4\} - \rho(r)\{Mo_5N_6\} - \rho(r)\{Si_2N_2\}$. It is used to explain the change of charge density when Mo_5N_6 is covered by Si-N layers. The yellow (cyan) region denotes the increase (decrease) of charge density. The isosurface is 8×10^{-3} e/bohr^3. The result shows that the Si-N layer strongly reconstructs the surface electronic structure of Mo_5N_6.

Table S1. The formation energies of bulk $MoSi_2N_4$ crystals with different stacking orders.

Stacking order	AA	AB	$A\bar{A}$	$A\bar{B}$	$A\bar{C}$	ABC
Formation energy (eV/atom)	-1.118	-1.132	-1.128	-1.118	-1.137	-1.519

Table S2. Calculated crystallographic parameters of bulk $MoSi_2N_4$ crystal with ABC-typed stacking order by the First-principles calculations. The experimentally obtained crystallographic parameters were also given for comparison.

$MoSi_2N_4$ (…ABCABC…), R3m (space group), a = b = 2.9179 Å (Exp. 2.94 Å), c = 30.3000 Å (Exp. 31.20 Å)				
Element site	X	Y	Z	Occupation
Mo1	0.33333	0.66667	0.13132	1.0
Si1	0	0	0.03222	1.0
Si2	0	0	0.23049	1.0
N1	0.66667	0.33333	0.01565	1.0
N2	0.66667	0.33333	0.24695	1.0
N3	0	0	0.08993	1.0
N4	0	0	0.17271	1.0

Table S3. The Young's modulus Y_{2D}, Poisson ratio v, thickness d and in-plane lattice constants a of $MoSi_2N_4(MoN)_n$ and $Mo_{n+1}N_{n+2}$ (n = 0 – 4). The Young's modulus and Poisson ratio of Mo_2N_3 were not given because of its mechanical instability.

Compound	Y_{2D} (GPa)	v	d (Å)	a (Å)
$MoSi_2N_4$	493.275	0.301	6.794	2.909
$MoSi_2N_4(MoN)_1$	490.558	0.249	9.774	2.913
$MoSi_2N_4(MoN)_2$	472.510	0.267	12.691	2.893
$MoSi_2N_4(MoN)_3$	489.142	0.263	15.556	2.887
$MoSi_2N_4(MoN)_4$	497.747	0.270	18.387	2.885
MoN_2	301.751	0.389	2.191	2.995
Mo_2N_3	---	---	5.119	2.903
Mo_3N_4	403.294	0.341	8.013	2.895
Mo_4N_5	415.174	0.291	10.852	2.898
Mo_5N_6	394.393	0.354	13.744	2.891

Table S4. The superconducting transition temperature T_c (K) of $MoSi_2N_4(MoN)_n$ (n = 1 – 4) and Mo_5N_6 calculated by screened Coulomb interaction μ* value of 0.1, electron-phonon coupling constants λ, and logarithmic average phonon frequencies ω_{log} (K).

Compound	T_c (K)	λ	ω_{log} (K)
$MoSi_2N_4(MoN)_1$	1.27	0.37	467.27
$MoSi_2N_4(MoN)_2$	7.22	0.57	363.55
$MoSi_2N_4(MoN)_3$	14.08	0.84	275.96
$MoSi_2N_4(MoN)_4$	9.02	0.61	370.43
Mo_5N_6	19.74	1.05	263.19

References

1. Perdew JP, Burke K and Ernzerhof M. Generalized Gradient Approximation Made Simple. *Phys Rev Lett* 1996; **77**: 3865-8.

2. Politano A and Chiarello G. Probing the Young's modulus and Poisson's ratio in

graphene/metal interfaces and graphite: a comparative study. *Nano Res* 2015; **8**: 1847-56.

3. Perdew JP and Zunger A. Self-interaction correction to density-functional approximations for many-electron systems. *Phys Rev B* 1981; **23**: 5048-79.

4. Baroni S, de Gironcoli S and Dal Corso A *et al*. Phonons and related crystal properties from density-functional perturbation theory. *Rev Mod Phys* 2001; **73**: 515-62.

5. McMillan WL. Transition Temperature of Strong-Coupled Superconductors. *Phys Rev* 1968; **167**: 331-44.

6. Allen PB and Dynes RC. Transition temperature of strong-coupled superconductors reanalyzed. *Phys Rev B* 1975; **12**: 905-22.

7. Ganin AY, Kienle L and Vajenine GV. Synthesis and characterization of hexagonal molybdenum nitrides. *J Solid State Chem* 2006; **179**: 2339-48.
